# Effect of the prolonged high-fat diet on the fatty acid metabolism in rat blood and liver

**DOI:** 10.1186/1476-511X-13-49

**Published:** 2014-03-16

**Authors:** Natalia V Zhukova, Tatyana P Novgorodtseva, Yulia K Denisenko

**Affiliations:** 1 A.V. Zhirmunsky Institute of Marine Biology of the Far East Branch of the Russian Academy of Sciences, Palchevskogo str., 17, 690041 Vladivostok, Russia; 2 School of Biomedicine, Far Eastern Federal University, Vladivostok, Russia; 3 Vladivostok Branch of the Far Eastern Center of Physiology and Pathology of Respiration of SB RAMN - Institute of Medical Climatology and Rehabilitative Treatment, Vladivostok, Russia

**Keywords:** High-fat diet, Fatty acids, Metabolism

## Abstract

**Background:**

Contradictory data on consequences of prolonged high-fat diet requires a detailed study of the influence of nutritional high-fat load mechanisms on the peculiarity of lipid metabolism in blood and liver. The present study was undertaken to investigate the fatty acid composition of polar and neutral lipids of the blood plasma, erythrocytes and liver in Wistar rats under the conditions of a prolonged high-fat diet.

**Methods:**

The study was conducted on 60 adult white male Wistar rats. The animals were fed on a high-fat diet consisted of the beef fat and cholesterol (19% and 2% of the total diet, respectively) up to 180 days. The fatty acid composition of the polar and neutral lipids of plasma, erythrocytes and liver were analyzed by the gas chromatography. Statistical data processing was performed by the methods of descriptive statistics with Statistica 6.0.

**Results:**

The prolonged unbalanced diet rich in cholesterol and saturated fatty acids resulted in compensatory biosynthesis of the fatty acids in the rat’s liver, the inhibition of synthesis of apoproteins and lipoproteins, disruption of the active transport of fatty acids to tissue cells. This launched the accumulation of 20:4n-6, 20:5n-3, 22:5n-3, and 22:6n-3 in the liver and blood plasma and deficiency of 18:2n-6, 20:5n-3 and 22:6n-3 in the erythrocytes.

**Conclusions:**

Adaptive adjustment of lipid metabolism un0064er conditions of the high-fat diet induced inhibition of the formation of lipoproteins (VLDL cholesterol) in the liver, compensatory synthesis of 18:1n-9, 20:5n-3, and 20:3n-6 with primary esterification of PUFA n-3 series to neutral lipids.

## Background

Alimentary factors, in particular saturated fats and cholesterol, can significantly alter the liver metabolic processes, affect lipid metabolism, biosynthesis of the fatty acids and the lipoproteins formation [1-3]. The results of the experimental and clinical observations have contributed to clarification of the role of fatty acids varying in the degree of saturation in the development of many diseases such as atherosclerosis, diabetes and steatohepatitis [4,5]. However, the concept of the relationship between increased consumption of the saturated fatty acids and the development of hyperlipidaemia with subsequent atherosclerosis and steatohepatitis is currently subject to criticism. Some studies have shown that consumption of triacylglycerol (TAG) rich in palmitic acid does not affect the levels of high density lipoprotein (HDL), cholesterol and total cholesterol in the blood [6]. There is evidence that excessive consumption of stearic acid lowers the level of low density lipoprotein (LDL) cholesterol and HDL cholesterol [7]. However, a decrease in the expression of the apo-B, E receptor of LDL at an increased consumption of saturated fats contributes to the accumulation of LDL and the development of hypercholesterolemia [8]. The experiments with rat model of lipid steatohepatitis have established that addition of saturated FA into the diet prevents liver necrosis and inflammation [8].

The regulatory effect of the alimentary fats on the lipid metabolism is realized at the level of modulation of the specific transcription factors expression involved in the metabolism of fatty acids - PGC-1β (peroxisome-proliferator-activated receptor-gamma coactivator 1 beta), HNF4 (hepatocyte nuclear factor 4), PPAR (peroxisome proliferator-activated receptor), and SREBP1 (sterol regulatory element-binding protein) [8-11]. At an excessive consumption of saturated fatty acids and simultaneous insufficient exogenous inflow of physiologically important essential fatty acids, the organism launches the particular mechanisms to activate the endogenous synthesis of monounsaturated and polyunsaturated fatty acids. This mechanism is mediated through the activation of the Δ9 desaturase, Δ6 desaturase and Δ5 desaturase and elongases. As a result, there occurs the activation of the compensatory *de novo* synthesis of monounsaturated and polyunsaturated fatty acids by hepatocytes. Modification of the fatty acids in the liver is a compensatory response that prevents deficiency of the physiologically important fatty acids. We may assume that in response to the high-fat diet in the liver two opposing (by its physiological role) processes are implemented: the accumulation of the dietary exogenous saturated lipids and the endogenous synthesis of monounsaturated and polyunsaturated fatty acids. The change of the lipid metabolism in the liver under the influence of the nutritional factors may be reflected in the transformation of the fatty acid composition in lipids of the blood plasma, cell membranes of the peripheral organs, erythrocytes in particular [12,13]. The fatty acid composition of erythrocyte membranes was used as a diagnostic criterion for assessment of different diseases [14,15], therefore the red blood cells were taken as a universal model for the study of pathological reactions.

Contradictory information requires a detailed study of the influence of nutritional high-fat load mechanisms on the peculiarity of lipid metabolism in blood and liver of rats. The aim of the present work was to study the fatty acid composition of polar and neutral lipids of the blood plasma, erythrocytes and liver in Wistar rats under the conditions of a prolonged high-fat diet.

## Methods

### Subjects

Experiments were carried out on 60 Wistar male rats. The following groups of animals were formed: Experiment 1 on rats fed the experimental diet for 30 days; Experiment 2, the experimental diet for 90 days; and Experiment 3, the experimental diet for 180 days. The control group 1 included 30 intact males kept on a standard vivarium diet for 30 days, the same for 90 days (control group 2), and the same for 180 days (control group 3). Each test group was compared with the respective control group, receiving the diet during the same time. The experimental diet contained beef fat (19% of the total diet) and cholesterol (2% of the total diet) [5] additionally to the standard ration.

The fatty acid composition of beef fat included saturated FA (12:0, 14:0, 16:0, and 18:0) to 66% of the total FA, monounsaturated FA (35%), and polyunsaturated fatty acids (2%). Animal euthanasia was carried out by decapitation under ether anesthesia in accordance with the requirements of the European Convention for the Protection of Experimental Animals, 86/609 EEC [16]. The euthanasia schedule was as follows: rats of the test group 1 and the respective control groups were euthanized on the 31st day; rats of the test group 2 and the respective control group 2 were euthanized on the 91st day; rats of the test group 3 and the respective control group 3 were euthanized on the 181st day. Morning fasting blood for research was taken from jugular vein of the rats after decapitation. The liver for fatty acid research was removed from rats after decapitation. The body weight of rats was recorded before and after the experiment.

### Lipid and fatty acid analysis

Lipid profile of the blood serum was studied on a biochemical analyzer FP-901 («Labsistems», Finland). Total cholesterol, triacylglyceride (TAG), and high-density lipoprotein (HDL) cholesterol were measured. The concentration of LDL cholesterol and very low density lipoprotein (VLDL) cholesterol were calculated by the Friedwald’s formula. The results are expressed in mmol/L.

$$\begin{array}{ll} \mathrm{LDL}\;\text{cholesterol}= & \text{Total}\;\text{cholesterol}-\mathrm{VLDL}\;\text{cholesterol}\\ & -\mathrm{HDL}\;\text{cholesterol}; \end{array}$$

VLDLcholesterol=TAG/2.2.

The index of atherogenicity (IA) was calculated as IA = (Total cholesterol – HDL)/HDL cholesterol.

Lipids were extracted using the solvent system chloroform – methanol, 1:2 (v/v), and then chloroform - methanol (1:1 v/v) and 0.9% sodium chloride were added until a complete phase separation is reached [17]. Separation of phospholipids and triacylglycerols from the lipids of the liver was performed by thin-layer chromatography (TLC) on silica gel using a solvent system, which consisted of hexan/diethyl ether/acetic acid (80:20:1, v/v). Fatty acid methyl esters (FAME) were obtained by a sequential treatment of the total lipids with 1% sodium methylate/methanol and 5% HCl/methanol according to Carreau and Dubacq [18] and purified by preparative silica gel thin-layer chromatography, using the silica gel plates developed in benzene. MEFA were analyzed on a Shimadzu GC-2010 (Japan) gas chromatograph equipped with a flame ionization detector, using a fused silica capillary column (Supelcowax-10, 30 m × 0.25 mm i. d. Supelco, Bellefonte, PA). Helium was used as a carrier gas at a linear velocity of 30 cms^–1^. The column temperature was 210°C, injector and detector temperatures were 250°C. Fatty acids were identified by a comparison with standard mixtures and equivalent chain length values [19]. The results were expressed in relative % of total FA.

### Statistical analysis

Statistical data processing was performed using the applied program statistics with Statistica 6.1. The differences in contents of the fatty acids between control groups and experimental groups of the rats were tested using a Student’s *t*-test. Differences were considered statistically significant at *P* < 0.05.

## Results and discussion

### Biometrics

The body weight of rats of the experimental group 1, which consumed the high-fat diet for 30 days, increased by 151 ± 11 g, which is by 84% higher compared to the initial weight. After 90 days of the experiment, the body weight of the animals of the experimental group 2 increased by 182 ± 6 g. In rats of the experimental group 3 (180 days of the high-fat diet), the body weight increased by 295 ± 65 g (2.6 times) of the initial weight. All values are significant at *P* < 0.001.

### Lipids in blood serum

High-fat load for 30 days resulted in increased levels of total cholesterol, triacylglycerols, LDL cholesterol, and VLDL cholesterol in the rats’ blood serum (Table 1). After 90 days of high fat load, we detected a reduced concentration of TAG and VLDL cholesterol and an increased content of HDL cholesterol in the blood of rats and decrease of IA. On the 180th day of high-fat diet, the total cholesterol and LDL cholesterol contents in rats’ blood serum were increased, while the level of VLDL cholesterol remained low. Dietary saturated fats modify cholesterol metabolism and increase significantly the concentration of HDL cholesterol and rise triglyceride accumulation in mouse after three weeks of high fat diet [20]. The obtained data demonstrate that alimentary fats exert a distinctly pronounced hyperlipidemic effect only on the 30th day of the experiment.

**Table 1:** Lipids of blood serum of rats at high-fat load

**Measure**	**High-fat load**					
	**30 days**		**90 days**		**180 days**	
	**Control**	**Experiment**	**Control**	**Experiment**	**Control**	**Experiment**
Total cholesterol, mmol/L	1.49 ± 0.04	3.68 ± 0.04***	1.56 ± 0.11	1.64 ± 0.08	1.59 ± 0.04	***2.82 ± 0.17
Triacylglycerols, mmol/L	1.19 ± 0.04	1.90 ± 0.06***	1.14 ± 0.01	***0.51 ± 0.05^xxx^	1.18 ± 0.04	***0.52 ± 0.08^xxx^
LDL cholesterol, mmol/L	0.29 ± 0.02	2.34 ± 0.11***	0.25 ± 0.04	***0.90 ± 0.12^xxx^	0.33 ± 0.02	***1.84 ± 0.17^x^
VLDL cholesterol, mmol/L	0.54 ± 0.02	0.85 ± 0.03***	0.55 ± 0.02	***0.23 ± 0.01^xxx^	0.64 ± 0.05	***0.22 ± 0.02^xxx^
HDLcholesterol, mmol/L	0.67 ± 0.04	0.28 ± 0.02***	0.69 ± 0.03	0.50 ± 0.08^xxx^	0.74 ± 0.06	0.75 ± 0.15^xxx^
Atherogenicity index	1.36 ± 0.15	11.87 ± 1.55***	1.49 ± 0.13	*2.9 ± 0.35^xxx^	1.50 ± 0.21	***2.44 ± 0.8^xxx^

Mean ± m, n = 10.

Note: Asterisks indicate significant differences of lipid contents between rats from experimental group and corresponding control group: *for *P* < 0.05; ***for *P* < 0.001. Asterisks indicate significant differences of lipid contents between rats from experimental group and preceding experimental group: ^x^for *P* < 0.05; ^xxx^for *P* < 0.001.

It is known that an increase in the blood lipid content after consumption of food is a normal physiological process of the digestive and lipid transport systems. The lipid level in blood achieves largest concentration in 30 minutes after consumption of food rich in fat. In our study, the fasting blood was taken from the rats in the morning. The minimum period of time between the last food consumption by test animals and the blood sampling was 12 hours. Accordingly, with this time interval, the changes in the content of atherogenic lipoproteins in the blood serum that were recorded on the 90th and the 180th days of the experiment are the result of a decrease in the synthesis of apoproteins and VLDL assembly in the liver. Ration rich in saturated fats may inhibit the VLDL synthesis in the liver [21,22]. Since VLDL transport TAG from the liver to organs and tissues in the body, insufficient VLDL synthesis causes accumulation of TAG in adipose and parenchymal tissues and in the liver.

Concentration of LDL in the blood is depends on the activity of their formation from lipoproteins of VLDL and the efficiency of LDL capture by apoB-100 receptors. Consequently, the accumulation of LDL cholesterol in the blood and interstitial fluid occurs at the blockage of the apoB-100 receptor-mediated endocytosis of LDL and at inhibition of hydrolysis of TAG contained in the VLDL [23]. An indicator of such disorders is an increase in the LDL concentration in the blood. The primary role of LDL is to transfer of PUFA to the cells in the form of non-polar esters of cholesterol. Blockage of active LDL capture by cells can lead to development of PUFA deficiency in the cells. A direct evidence of such disorder can be an increased circulation of lipids rich in unsaturated FA in blood at a simultaneous deficit of these FA in cell membranes. In order to confirm this assumption and to establish the features of lipid exchange during a prolonged nutrition load, we have studied the fatty acid composition of polar and neutral lipids in blood plasma and in red blood cells of rats in the dynamics of the impact of high fat diet.

### Fatty acids of blood plasma and erythrocytes

It was found that the levels of 14:0 and 15:0 in the pool of phospholipids (PL), TAG and sterol esters (ES) in the rats’ blood were on the 30th day of the high-fat load increased (Table 2). As compared with the control group, the share of 18:0 was decreased in TAG and increased in PL. An increase of 18:1n-9 content was revealed in the erythrocytes. A significant decrease in the relative contents of 18:2n-6 was found in the TAG and PL (*P* < 0.001). The TAG and PL had low level of 20:3n-9 and high contents of 20:5n-3 and 22:5n-3 (Table 2). A significant decrease in the share of 20:4n-6 was detected in all studied lipid fractions of the blood plasma (*P* < 0.001). The dynamics of these fatty acids in erythrocytes on the 30th day of the nutritional stress was characterized by an increase in the shares of 14:0, 18:0, 20:4n-6 and 22:4n-6 and a decrease in the levels of 18:2n-6 and 22:6n-3.

**Table 2:** The content of fatty acids (wt%) in plasma and erythrocytes of rats at a high-fat load

**Fatty acid**	**Source**	**High-fat load**					
		**30 days**		**90 days**		**180 days**	
		**Control**	**Experiment**	**Control**	**Experiment**	**Control**	**Experiment**
14:0	Erythrocyte	0.52 ± 0.04	*0.83 ± 0.08 / **1.6↑**	0.48 ± 0.04	0.5 ± 0.06	0.50 ± 0.03	***1.01 ± 0.12 / **2↑**
	Plasma PL	0.50 ± 0.04	***1.22 ± 0.20 / **2.2↑**	0.62 ± 0.04	0.57 ± 0.05	0.60 ± 0.07	**0.37 ± 0.04 / **1.6↓**
	Plasma TAG	1.43 ± 0.10	*1.71 ± 0.10 / **1.2↑**	1.50 ± 0.10	1.53 ± 0.15	1.48 ± 0.10	*0.87 ± 0.11 / **1.7↓**
	Plasma ES	1.05 ± 0.06	1.23 ± 0.01	0.95 ± 0.09	0.84 ± 0.06	1.10 ± 0.05	***0.42 ± 0.05 / **2.5↓**
15:0	Erythrocyte	0.52 ± 0.04	0.58 ± 0.07	0.72 ± 0.06	**0.43 ± 0.02 / **1.7↓**	0.76 ± 0.07	*0.56 ± 0.04 / **1.4↓**
	Plasma PL	0.75 ± 0.06	***1.33 ± 0.07 / **1.7↑**	0.72 ± 0.05	***0.45 ± 0.02 / **1.6↓**	0.68 ± 0.05	***0.40 ± 0.01 / **1.7↓**
	Plasma TAG	0.92 ± 0.08	***1.55 ± 0.07 / **1.6↑**	0.90 ± 0.10	0.86 ± 0.05	0.94 ± 0.07	**0.52 ± 0.08 / **1.8↓**
	Plasma ES	0.80 ± 0.02	***1.36 ± 0.09 / **1.7↑**	0.84 ± 0.04	***0.41 ± 0.03 / **2↓**	0.93 ± 0.02	***0.35 ± 0.01 / **2.6↓**
16:0	Erythrocyte	23.72 ± 0.84	24.38 ± 0.57	24.12 ± 0.68	***28.6 ± 0.5 / **1.2↑**	24.72 ± 0.74	**27.6 ± 1.4 / **1.1↑**
	Plasma PL	28.50 ± 0.33	27.68 ± 0.56	26.50 ± 0.43	25.64 ± 0.83	28.70 ± 0.30	27.02 ± 0.36
	Plasma TAG	28.16 ± 2.24	26.42 ± 0.68	27.60 ± 2.00	24.23 ± 1.66	28.86 ± 1.80	**22.2 ± 0.52 / **1.3↓**
	Plasma ES	17.32 ± 3.49	15.67 ± 1.66	17.18 ± 1.00	***11.47 ± 0.43 / **1.5↓**	17.00 ± 0.09	***11.32 ± 0.12 / **1.5↓**
16:1n-7	Erythrocyte	1.90 ± 0.25	1.83 ± 0.12	1.88 ± 0.22	***0.83 ± 0.05 / **2.3↓**	2.04 ± 0.30	2.04 ± 0.21
	Plasma PL	1.2 ± 0.1	***3.31 ± 0.40 / **2.7↑**	1.1 ± 0.2	0.91 ± 0.07	1.1 ± 0.6	1.17 ± 0.14
	Plasma TAG	6.57 ± 0.34	*8.67 ± 0.14 / **1.3↑**	7.56 ± 0.30	***2.53 ± 0.27 / **3↓**	8.10 ± 0.46	*2.72 ± 0.39 / **3↓**
	Plasma ES	5.35 ± 0.59	***8.14 ± 0.77 / **1.6↑**	5.20 ± 0.40	*3.28 ± 57.14 / **1.6↓**	5.15 ± 0.90	4.75 ± 0.19
18:0	Erythrocyte	10.10 ± 0.50	**14.68 ± 0.48 / **1.4↑**	10.7 ± 0.45	***17.3 ± 0.57 / **1.7↑**	9.9 ± 0.40	***13.9 ± 1.77 / **1.4↑**
	Plasma PL	19.00 ± 0.37	*21.67 ± 0.94 / **1.1↑**	19.40 ± 0.67	***23.23 ± 0.14 / **1.2↑**	18.70 ± 0.70	18.97 ± 0.72
	Plasma TAG	5.00 ± 0.03	*3.00 ± 0.16 / **1.6↓**	4.80 ± 0.10	***8.72 ± 0.91 / **1.8↑**	5.10 ± 0.30	4.92 ± 0.34
	Plasma ES	3.75 ± 0.54	3.72 ± 0.46	3.45 ± 0.94	3.35 ± 0.22	3.70 ± 0.50	3.15 ± 0.56
18:1n-9	Erythrocyte	7.66 ± 0.57	**11.45 ± 1.22 / **1.5↑**	7.51 ± 0.70	**10.5 ± 0.31 / **1.4↑**	8.16 ± 0.67	**11.2 ± 1.38 / **1.4↑**
	Plasma PL	6.77 ± 0.41	7.67 ± 0.28	6.14 ± 0.50	6.91 ± 2.09	6.35 ± 0.60	*9.52 ± 0.34 / **1.5↑**
	Plasma TAG	25.27 ± 2.26	26.5 ± 2.36	27.70 ± 2.20	30.66 ± 1.87	26.10 ± 1.20	***36.55 ± 1.14 / **1.4↑**
	Plasma ES	22.30 ± 3.7	***11.5 ± 0.9 / **2↓**	21.64 ± 2.0	***36.8 ± 2.7 / **1.7↑**	21.30 ± 1.70	***46.5 ± 0.8 / **2.2↑**
18:2n-6	Erythrocyte	14.02 ± 0.64	***9.78 ± 0.23 / **1.4↓**	13.12 ± 0.64	12.6 ± 0.4	14.62 ± 0.84	**11.3 ± 0.97 / **1.3↓**
	Plasma PL	18.50 ± 0.6	18.85 ± 1.05	18.9 ± 0.60	18.12 ± 1.21	17.8 ± 0.50	18.30 ± 0.20
	Plasma TAG	19.62 ± 2.86	***15.3 ± 1.1 / **1.2↓**	20.20 ± 2.00	20.23 ± 1.40	18.82 ± 1.80	*17.12 ± 0.16 / **1.1↓**
	Plasma ES	19.25 ± 1.19	19.17 ± 1.35	20.25 ± 1.10	21.08 ± 0.96	18.40 ± 1.00	***13.12 ± 0.2 / **1.4↓**
20:3n-9	Erythrocyte	0.35 ± 0.12	tr	0.27 ± 0.10	0.17 ± 0.03	0.31 ± 0.10	***0.95 ± 0.09 / **3↑**
	Plasma PL	0.55 ± 0.02	***0.27 ± 0.02 / **2↓**	0.54 ± 0.10	***0.25 ± 0.09 / **2↓**	0.62 ± 0.12	0.65 ± 0.02
	Plasma TAG	0.72 ± 0.05	***0.30 ± 0.01 / **2.4↓**	0.77 ± 0.08	0.73 ± 0.12	0.74 ± 0.06	***1.45 ± 0.34/ **2↑**
	Plasma ES	0.96 ± 0.17	0.83 ± 0.31	0.94 ± 0.14	***0.45 ± 0.05 / **2.1↓**	0.89 ± 0.20	0.75 ± 0.17
20:3n-6	Erythrocyte	0.60 ± 0.13	0.88 ± 0.09	0.62 ± 0.10	**1.23 ± 0.06 / **2↑**	0.70 ± 0.10	0.9 ± 0.08
	Plasma PL	1.77 ± 0.39	1.38 ± 0.12	1.82 ± 0.30	2.05 ± 0.14	2.10 ± 0.70	*3.05 ± 0.29
	Plasma TAG	0.77 ± 0.38	***0.27 ± 0.20 / **2.8↓**	0.67 ± 0.30	0.51 ± 0.06	0.70 ± 0.30	0.72 ± 0.19
	Plasma ES	0.42 ± 0.06	*0.66 ± 0.08 / **1.5↑**	0.52 ± 0.10	0.55 ± 0.06	0.48 ± 0.09	0.47 ± 0.02
20:4n-6	Erythrocyte	6.78 ± 1.70	***21.27 ± 1.92 / **3.1↑**	6.80 ± 1.20	***16.3 ± 0.5 / **2.4↑**	6.90 ± 1.72	***15.9 ± 0.94 / **2.3↑**
	Plasma PL	12.2 ± 0.72	***7.56 ± 0.62 / **1.7↓**	12.8 ± 0.80	13.66 ± 0.72	13.0 ± 0.80	**9.80 ± 0.60 / **1.3↓**
	Plasma TAG	2.45 ± 0.65	***0.87 ± 0.16 / **2.7↓**	2.50 ± 0.60	2.38 ± 0.25	2.20 ± 0.50	2.11 ± 0.32
	Plasma ES	28.73 ± 7.34	**18.84 ± 2.73 / **1.5↓**	30.54 ± 3.30	***10.96 ± 0.86 / **2.8↓**	28.50 ± 2.40	***10.55 ± 0.90 / **2.7↓**
20:5n-3	Erythrocyte	0.90 ± 0.13	0.73 ± 0.09	0.86 ± 0.10	*0.58 ± 0.05	0.93 ± 0.12	**0.68 ± 0.05
	Plasma PL	0.42 ± 0.13	***1.55 ± 0.18 / **3.7↑**	0.48 ± 0.10	0.62 ± 0.11	0.50 ± 0.10	0.50 ± 0.10
	Plasma TAG	0.70 ± 0.03	***1.17 ± 0.11 / **1.6↑**	0.72 ± 0.08	*0.92 ± 0.13 / **1.3↑**	0.73 ± 0.07	**0.95 ± 0.05 / **1.3↑**
	Plasma ES	0.70 ± 0.20	tr	0.75 ± 0.10	***1.24 ± 0.12 / **1.7↑**	0.80 ± 0.20	0.95 ± 0.11
22:4n-6	Erythrocyte	0.58 ± 0.07	**1.58 ± 0.09 / **3↑**	0.53 ± 0.07	**0.85 ± 0.05 / **1.6↑**	0.56 ± 0.06	0.66 ± 0.08
	Plasma PL	0.15 ± 0.05	*0.33 ± 0.08 / **2↑**	0.14 ± 0.07	***0.48 ± 0.06 / **3.2↑**	0.15 ± 0.05	0.15 ± 0.04
	Plasma TAG	0.30 ± 0.01	0.27 ± 0.01	0.20 ± 0.05	tr	0.31 ± 0.06	***0.61 ± 0.02 / **2↑**
	Plasma ES	0.24 ± 0.04	tr	tr	tr	tr	tr
22:5n-6	Erythrocyte	0.23 ± 0.15	*0.55 ± 0.05 / **2.5↑**	0.22 ± 0.10	***0.17 ± 0.03 / **1.3↓**	0.21 ± 0.12	***0.53 ± 0.13 / **2.5↑**
	Plasma PL	0.20 ± 0.01	tr	0.23 ± 0.05	***0.55 ± 0.01 / **2.4↑**	0.15 ± 0.05	tr
	Plasma TAG	tr	tr	tr	0.20 ± 0.01	tr	tr
	Plasma ES	0.10 ± 0.07	tr	tr	tr	tr	0.20 ± 0.01
22:5n-3	Erythrocyte	1.32 ± 0.16	1.62 ± 0.27	1.22 ± 0.14	1.38 ± 0.09	1.12 ± 0.17	1.15 ± 0.27
	Plasma PL	0.62 ± 0.13	***0.92 ± 0.10 / **1.5↑**	0.60 ± 0.15	0.61 ± 0.08	0.67 ± 0.20	0.65 ± 0.06
	Plasma TAG	0.50 ± 0.02	***1.05 ± 0.13 / **2↑**	0.64 ± 0.08	0.74 ± 0.11	0.54 ± 0.06	***0.25 ± 0.07 / **2↓**
	Plasma ES	0.15 ± 0.05	tr	tr	0.36 ± 0.09	tr	0.17 ± 0.02
22:6n-3	Erythrocyte	5.53 ± 0.12	*3.93 ± 0.11 / **1.4↓**	4.53 ± 0.16	**2.22 ± 0.08 / **2↓**	5.38 ± 0.17	***2.85 ± 0.53 / **1.9↓**
	Plasma PL	3.22 ± 0.47	4.45 ± 0.50	3.00 ± 0.70	3.27 ± 0.24	3.80 ± 0.80	4.27 ± 0.35
	Plasma TAG	1.45 ± 0.13	*2.93 ± 0.09 / **2↑**	1.85 ± 0.40	2.46 ± 0.33	1.10 ± 0.10	***3.25 ± 0.32 / **3↑**
	Plasma ES	1.65 ± 0.38	1.95 ± 0.11	1.35 ± 0.30	1.14 ± 0.12	1.74 ± 0.28	***0.87 ± 0.14 / **2↓**

Mean ± m, n = 10.

Note: TAG, triacylglycerols; PL, phospholipids; ES, esters of sterols; tr, traces less than 0.1%. Asterisks indicate significant differences of fatty acid contents between rats from experimental group and corresponding control group: *for *P* < 0.05; **for P < 0.01, ***for *P* < 0.001. The numbers in boldface after the slash indicate the change of the value for the experimental group relative to that of the control group in the number of times. The arrow **↓** indicates that the value decreased. The arrow **↑** indicates that the value increased.

On the 90th day of the experiment the contents of the fatty acids 15:0 and 20:3n-9 in PL and ES in the blood plasma was decreased. The level of 18:0 was increased in the PL and TG of the blood plasma and erythrocytes. The share of 18:1n-9 was increased in the erythrocytes and in ES of the blood plasma. A decreased content of 20:4n-6 was identified in plasma ES, whereas in blood cells the level of 20:4n-6 and its predecessor 20:3n-6 were increased. In the erythrocytes, a fall of the level of 20:5n-3 was revealed on the background of its increase in the ES and TG of the blood plasma. The relative contents of 22:5n-3 and 22:6n-3 in lipids of the blood plasma did not differ from those in the control group. In blood cells, the levels of 22:5n-6 and 22:6n-3 were decreased, while the 22:4n-6 level was increased. Therefore, the vector of changes in the PUFA composition of plasma lipids and red blood cells had on the 90th day of the experiment a reciprocal direction. An increase in the amounts of 20:5n-3, 22:6n-3, and 22:5n-6 in the blood plasma was revealed with a simultaneous deficiency of these fatty acids in cell membranes.

After 180 days of the high-fat diet, the FA profile of plasma lipids in rats was characterized by a decrease in the amounts of 14:0 and 15:0. The PUFA pool (except PL) was depleted in 18:2n-6. The level of 18:1n-9 remained elevated. A deficiency of 20:4n-6 was detected in ES. The contents of 20:5n-3 and 22:4n-6 in TAG was increased. The level of 22:6n-3 remained unchanged in PL, increased in TAG and decreased in ES of the plasma. Paradoxical at first glance phenomenon of increasing levels of C20 and C22 PUFA in lipids of the blood plasma distinctly fits into the concept on the disorder of FA transport. Violation of the receptor capture of the lipoproteins by cells leads to a PUFA deficiency in cell membranes and to compensatory activation of the passive transport of the saturated FAs. Indeed, the modification of the FA composition of erythrocytes on the 180th day of high-fat diet consumption was characterized by increased levels of saturated acids (14:0, 16:0, 18:0), lower contents of 18:2n-6, 20:5n-3, and 22:6n-3. We can suggest that the indicator of deficit of the fatty acids n-6 and n-3 in cells may be an increased level of Mead acid (20:3n-9) in the erythrocytes’ membranes.

### Fatty acids of the rat liver

An increase of the content of 16:0 and a decrease in the content of 18:0 were revealed in the FA composition of the rat liver after 30 days of the high-fat load (Table 3). The elevated level of 16:0 in the liver was due to the peculiarity of the experimental diet that was enriched with this FA. Indeed, the dietary saturated FA and cholesterol, i.e. effectors of expression of the transcription factors (sterol regulatory element-binding protein, SREBP) induce the synthesis of 16:0 and TAG and inhibit VLDL production, thus causing steatosis of the liver [7]. The increased content of 18:1n-9 was accompanied by a decrease in the concentration of 18:0; this might indicate activation of Δ9-desaturase, which performs metabolic conversion in the reaction of 18:0 → 18:1n-9. Moreover, there occurred a simultaneous increase in the contents of 18:3n-6 and 18:4n-3 and decrease in the contents of 20:4n-6, 20:5n-3, 22:5n-3, and 22:6n-3.

**Table 3:** Fatty acid composition (wt%) of total lipids of rat liver at a 30 day high-fat load

**Fatty acid**	**Control**	**Experiment**
14:0	0.60 ± 0.09	0.53 ± 0.02
15:0	0.36 ± 0.05	0.33 ± 0.02
16:0	19.08 ± 0.59	*25.10 ± 1.45
18:0	10.42 ± 1.04	**5.80 ± 0.45
18:1n-9	16.32 ± 1.13	***33.90 ± 0.9
18:1n-7	2.76 ± 0.22	3.40 ± 0.32
18:2n-6	15.08 ± 1.05	*13.20 ± 0.63
18:3n-6	0.15 ± 0.09	*0.38 ± 0.02
18:3n-3	0.32 ± 0.02	0.27 ± 0.09
18:4n-3	0.02 ± 0.02	***0.15 ± 0.05
20:2n-6	0.14 ± 0.02	***0.65 ± 0.05
20:3n-6	0.82 ± 0.04	**1.33 ± 0.07
20:4n-6	11.72 ± 1.05	***4.20 ± 0.26
20:5n-3	1.25 ± 0.05	***0.50 ± 0.02
22:4n-6	0.10 ± 0.06	0.23 ± 0.09
22:5n-3	1.54 ± 0.10	***0.75 ± 0.05
22:6n-3	10.34 ± 0.30	***4.53 ± 0.43

Mean ± m, n = 10.

Note: Asterisks indicate significant differences of fatty acid contents between rats from experimental group and control: *for *P* < 0.05; **for *P* < 0.01; ***for *P* < 0.001.

After 90 days of the experiment, the content of 14:0 in ES of the liver was increased. The level of 16:0 was decreased in all lipid fractions (Table 4). The level of 18:0 was increased in PL and decreased in ES. The metabolic conversions of the PUFA after 90 days of the experiment led to increase in the contents of 18:1n-9 in PL, TAG, and ES; of 18:3n-3 in TAG and EC; of 18:2n-6 in TAG, ES, and PL; of 20:5n-3 in ES, and 20:3n-6 in PL and TAG. Decreased amounts of 20:4n-6 and 20:3n-9 were revealed in all lipid fractions of the rat liver. The amount of 22:5n-3 was increased in TAG. The maintenance of the levels of C20 and C22 PUFA n-3 and n-6 plays an important role in adaptation of the organism to their temporary alimentary limitation.

**Table 4:** The content of fatty acids in the liver of rats at a high-fat load

**Fatty acid**	**Lipids**	**High-fat load**			
		**90 days**		**180 days**	
		**Control**	**Experiment**	**Control**	**Experiment**
14:0	PL	0.28 ± 0.04	0.26 ± 0.03	0.22 ± 0.04	0.20 ± 0.04
	TAG	0.86 ± 0.09	*0.63 ± 0.03 / **1.3↓**	0.85 ± 0.06	*0.65 ± 0.05 / **1.3↓**
	ES	0.58 ± 0.12	***1.2 ± 0.4 / **2↑**	0.61 ± 0.14	***0.27 ± 0.03 / **2.1↓**
16:0	PL	20.48 ± 0.47	***17.56 ± 1.09 / **1.2↓**	20.51 ± 0.33	19.50 ± 0.27
	TAG	26.47 ± 0.56	***16.63 ± 0.43 / **1.6↓**	26.47 ± 0.56	***19.14 ± 0.47 / **1.3↓**
	ES	49.65 ± 2.51	***14.1 ± 4.1 / **3.5↓**	51.20 ± 1.95	***11.2 ± 0.61 / **4↓**
18:0	PL	18.97 ± 0.67	***23.73 ± 0.81 / **1.2↑**	18.77 ± 0.67	19.8 ± 0.56
	TAG	2.45 ± 0.05	3.3 ± 0.2	2.21 ± 0.01	2.4 ± 0.17
	ES	6.18 ± 0.38	*4.7 ± 1.2 / **1.5↓**	6.27 ± 0.34	***3.68 ± 0.43 / **2↓**
18:1n-9	PL	4.71 ± 0.19	***6.0 ± 0.2 / **1.2↑**	3.51 ± 0.24	***8.64 ± 0.33 / **2↑**
	TAG	28.57 ± 3.06	**34.56 ± 1.09 / **1.2↑**	27.64 ± 1.24	***46.06 ± 1.28 / **2↑**
	ES	15.57 ± 1.61	***54.03 ± 4.66 / **3.6↑**	15.78 ± 1.54	***57.7 ± 3.49 / **3.8↑**
18:2n-6	PL	13.4 ± 0.44	***18.20 ± 0.20 / **1.4↑**	13.2 ± 0.27	**14.90 ± 0.40 / **1.1↑**
	TAG	18.3 ± 0.7	***27.86 ± 0.61 / **1.5↑**	18.0 ± 0.2	*16.5 ± 0.22 / **1.1↓**
	ES	6.85 ± 0.70	***15.16 ± 1.5 / **2↑**	6.52 ± 0.21	**8.94 ± 0.19 / **1.3↑**
18:3n-3	PL	0.15 ± 0.03	0.10 ± 0.05	0.12 ± 0.05	0.15 ± 0.05
	TAG	0.72 ± 0.05	*1.04 ± 0.08 / **1.6↑**	0.69 ± 0.01	0.71 ± 0.15
	ES	0.52 ± 0.12	***1.1 ± 0.10 / **2.3↑**	0.49 ± 0.09	*0.85 ± 0.19 / **2↑**
20:3n-9	PL	0.40 ± 0.09	0.25 ± 0.05	0.36 ± 0.06	0.27 ± 0.08
	TAG	0.34 ± 0.08	tr	0.31 ± 0.08	0.32 ± 0.06
	ES	tr	tr	tr	0.28 ± 0.02
20:3n-6	PL	1.35 ± 0.09	***2.96 ± 0.23 / **2.2↑**	1.51 ± 0.08	***2.88 ± 0.23 / **2.2↑**
	TAG	0.46 ± 0.15	*1.03 ± 0.2 / **2↑**	0.39 ± 0.06	0.5 ± 0.06
	ES	0.38 ± 0.16	tr	0.78 ± 0.11	***0.3 ± 0.05 / **2.6↓**
20:4n-6	PL	21.86 ± 0.68	*17.23 ± 1.46 / **1.2↓**	22.91 ± 0.21	*17.5 ± 1.56 / **1.2↓**
	TAG	3.11 ± 0.36	***1.1 ± 0.05 / **3↓**	2.59 ± 0.51	***0.78 ± 0.07 / **3.8↓**
	ES	4.90 ± 0.34	***1.15 ± 0.28 / **4↓**	5.10 ± 0.21	***1.2 ± 0.15 / **4↓**
20:5n-3	PL	0.57 ± 0.05	0.6 ± 0.05	0.54 ± 0.05	0.69 ± 0.11
	TAG	0.97 ± 0.13	0.7 ± 0.05	1.07 ± 0.17	***0.40 ± 0.07 / **2.3↓**
	ES		***0.3 ± 0.03 / **1.6↑**		0.31 ± 0.1
22:4n-6	PL	0.35 ± 0.05	***0.10 ± 0.01 / **3↓**	0.34 ± 0.05	*0.23 ± 0.02 / **1.5↓**
	TAG	0.38 ± 0.03	0.33 ± 0.03	0.36 ± 0.01	0.48 ± 0.26
	ES	0.60 ± 0.01	tr	0.60 ± 0.01	tr
22:5n-3	PL	1.03 ± 0.08	1.13 ± 0.08	1.04 ± 0.086	0.83 ± 0.08
	TAG	0.98 ± 0.09	***2.13 ± 0.08 / **2↑**	0.90 ± 0.10	0.85 ± 0.16
	ES	0.30 ± 0.01	^***^0.10 ± 0.01 / **3↑**	0.34 ± 0.04	tr
22:6n-3	PL	8.30 ± 0.46	8.50 ± 0.45	7.95 ± 0.16	8.6 ± 0.97
	TAG	3.08 ± 0.37	4.0 ± 0.3	3.21 ± 0.21	*2.8 ± 0.10 / **1.1↓**
	ES	0.50 ± 0.03	0.63 ± 0.18	0.56 ± 0.07	0.61 ± 0.07

Mean ± m, n = 10.

TAG, triacylglycerols; PL, phospholipids; ES, esters of sterols; tr, traces less than 0.1%. Asterisks indicate significant differences of fatty acid contents between rats from experimental group and corresponding control group: *for *P* < 0.05; **for *P* < 0.01; ***for *P* < 0.001. The numbers in boldface after the slash indicate the change of the value for the experimental group relative to that of the control group in the number of times. The arrow **↓** indicates that the value decreased. The arrow **↑** indicates that the value increased.

On the 180th day of the high-fat load, the levels of 14:0, 16:0 and 18:0 in the ES of the liver were reduced. The concentrations of 14:0 and 16:0 in TAG were decreased. Low concentrations of 22:4n-6 in PL and of 18:2n-6 and 20:5n-3 in TAG were found in rats’ liver. The deficiency of 20:4n-6 was detected in all the studied fractions of lipids. The content of 18:1n-9 was increased in the ES, PL and TAG. The high level of 18:2n-6 in ES and PL was maintained, but was not as distinctly pronounced as that on the 90th day of the experiment. The amount of 18:3 n-3 remained high only in the ES. The results of our study on the FA composition of the rat’s liver are well comparable to those that have been obtained in the analysis of FA levels in lipids of the rats’ blood plasma. The data on distribution of 20:5n-3 and 20:3n-6 were an only exception. So, in the lipids of the blood plasma, 20:5n-3 was identified mainly in TAG and ES, while 20:3n-6 was detected in the PL. Such a redistribution of the FA between the polar and neutral lipids is necessary to maintain homeostasis of 20:5n-3, which is precursor of oxylipins possessing vasoactive and anti-inflammatory properties [24,25]. The FA composition of the blood plasma lipids can be recognized as an indicator of the metabolic transformations of fatty acids in the liver. Similarity of the changes in the FA composition that occur in the liver and in the blood plasma allows us to confirm that the FA composition of the blood plasma may be recognized as an indicator of the metabolic alterations in fatty acids of the liver.

It is known that syntheses of TAG, PL and ES occur in the body from the two main pools: from free FA of plasma and from FA synthesized *de novo* in the liver [9]. The alimentary deficiency of the essential PUFA in the high-fat diet launches the compensatory synthesis of endogenous FA [8,9]. Effect of the alimentary lipids on the gene expression represents an adaptive response to changes in the quantity and type of the consumed fat. The direct effect of the saturated FA is due to the interaction with the transcription factors. The high-fat diet rich in saturated fatty acids (14:0, 16:0, and 18:0) induces the expression of the lipogenetic genes (PGC-1β, SREBP 1c, and other) responsible for the *de novo* synthesis of FA and their elongation, but also inhibits the assembly of VLDL-TAG through suppression of the synthesis of mRNA apo-proteins [6-8]. Correspondingly, a high-fat load on the liver launches two physiologically opposite processes; accumulation of exogenous saturated FA and endogenous biosynthesis of monounsaturated FA and PUFA.

Thus, the prolonged unbalanced diet rich in cholesterol and saturated FA promotes the compensatory synthesis of polyunsaturated fatty acids; this is confirmed by an increase in concentrations of dihomo-γ-linolenic acid 20:3n-6, eicosapentaenoic acid 20:5n-3 and arachidonic acid 20:4n-6 as well as oleic acid 18:1n-9 in lipids of the blood plasma and the liver. At the same time, because of the disorder of the active apoB-100 receptor transport of fatty acids contained in LDL, polyunsaturated fatty acids synthesized *de novo* in the liver are not captured by the peripheral organs’ cells. This fact is evidenced by the experimentally proved deficiency of PUFAs n-3 and n-6 in erythrocytes at distinct accumulation of lipid fractions in the blood plasma. An indicator of disorders of the blockade of lipoproteins, transporting polyunsaturated fatty acids from the liver to other organs is an increased level of the LDL cholesterol in the blood serum. The data obtained in our experiment opens an opportunity to understand the physiological role of the nutritional factors and some mechanisms of development of many alimentary-dependent diseases, including atherosclerosis, diabetes, and steatohepatitis.

## Abbreviations

FA: Fatty acid; HDL: High density lipoproteins; ES: Sterol esters; LDL: Low density lipoproteins; PL: Phospholipids; PUFA: Polyunsaturated fatty acid; TAG: Triacylglycerols; VLDL: Very low density lipoproteins.

## Competing interests

The authors declare that they have no competing interests.

## Authors’ contributions

NVZ performed fatty acid analysis, participated in writing and translating and has responsibility for the final content. TAG, YKK were responsible for the initial conception and design of the study and participated in discussion of the results and writing the manuscript. All the authors read and approved the final version of the manuscript.
